# Action and function of Wnt/β-catenin signaling in the progression from chronic hepatitis C to hepatocellular carcinoma

**DOI:** 10.1007/s00535-016-1299-5

**Published:** 2016-12-29

**Authors:** Wenhui Wang, Qiuwei Pan, Gwenny M. Fuhler, Ron Smits, Maikel P. Peppelenbosch

**Affiliations:** Department of Gastroenterology and Hepatology, Erasmus University Medical Center, ’s Gravendijkwal 230, 3015 CE Rotterdam, Netherlands

**Keywords:** Hepatocellular carcinoma, Wnt/β-catenin signaling, Hepatitis C virus

## Abstract

Hepatitis C virus (HCV) infection is one of the leading causes of hepatocellular carcinoma (HCC) worldwide but the mechanistic basis as to how chronic HCV infection furthers the HCC process remains only poorly understood. Accumulating evidence indicates that HCV core and nonstructural proteins provoke activation of the Wnt/β-catenin signaling pathway, and the evidence supporting a role of Wnt/β-catenin signaling in the onset and progression of HCC is compelling. Convincing molecular explanations as to how expression of viral effectors translates into increased activity of the Wnt/β-catenin signaling machinery are still largely lacking, hampering the design of rational strategies aimed at preventing HCC. Furthermore, how such increased signaling is especially associated with HCC oncogenesis in the context of HCV infection remains obscure as well. Here we review the body of contemporary biomedical knowledge on the role of the Wnt/β-catenin pathway in the progression from chronic hepatitis C to cirrhosis and HCC and explore potential hypotheses as to the mechanisms involved.

## Introduction

Hepatitis C virus (HCV) is estimated to infect up to 2% of the global population (around 180 million people worldwide) [1], with approximately 3 million to 4 million new infections each year [2, 3]. Following infection, 60–80% of affected individuals eventually develop chronic hepatitis [4]. After around 10 years of infection, 5–10% of these chronically infected patients develop cirrhosis [5]. In addition to the high mortality associated with advanced cirrhosis per se, annually another 2.0–6.6% of cirrhotic patients with HCV infection develop hepatocellular carcinoma (HCC) [6, 7]. Understanding the details as to how HCV infection can promote the HCC process is thus of critical importance for the rational design of novel avenues aimed at the prevention and treatment of HCC.

Distinct from hepatitis B virus (HBV), a DNA virus that can integrate into the human genome and thus directly provoke genomic alterations potentially leading to cancer [8], HCV is a RNA virus lacking a DNA intermediate phase in its life cycle, and therefore its infection of liver cells is not associated with damage to the host genetic material per se [9]. Hence the tumor-promoting potential of HCV derives from indirect interaction with the hepatocyte genome. It thus appears that HCV has specific properties that promote further hepatocyte transformation.

The Wnt/β-catenin pathway is an attractive candidate to mediate the HCV-specific effects leading to hepatocyte oncogenic transformation. Activation of this pathway clearly contributes to hepatocarcinogenesis as indicated by the detection of recurrent genetic mutations of Wnt/β-catenin signaling pathway components in HCC that appear especially frequent in HCV-related tumors. HCV-derived viral proteins appear to be capable of autonomous activation of Wnt/β-catenin signaling, although the underlying molecular mechanisms remain poorly understood. Here we explore potential hypotheses explaining these effects and summarize documented interactions of Wnt/β-catenin signaling components in HCC patients with HCV infection. We propose that the Wnt/β-catenin signaling pathway constitutes a rational target for the prevention and treatment of HCV-associated HCC.

## Wnt/β-catenin signaling

Wnt/β-catenin signaling is a pivotal morphogenetic pathway and accordingly is associated with a host of physiological and pathophysiological processes, including embryonic patterning, cell proliferation, cell differentiation, angiogenesis, and especially cancer [10–12]. Wnt signaling is initiated by binding of Wnt ligands to their cognate receptors. These Wnt ligands are 40-kDa cysteine-rich glycoproteins [13], which following synthesis and primary glycosylation on the endoplasmic reticulum are palmitoylated by Wnt acyltransferase porcupine protein in the Golgi apparatus. Secretion of Wnts then requires evenness interrupted/Wntless/G-protein-coupled receptor 177, which shuttles palmitoylated Wnts to the plasma membrane, where they are released by the cell and initiate autocrine or paracrine signaling. Hitherto, 19 Wnts have been identified in the human genome [14], and because annotation of *Homo sapiens* DNA is now quite complete, it is unlikely further Wnt paralogues will be discovered. Wnts can provoke different modes of cellular signaling, either mediated by β-catenin or independent of this protein. According to the dependence on β-catenin for provoking cellular effects, Wnts are classified into canonical (β-catenin-dependent) and noncanonical (β-catenin-independent) subgroups [15, 16]. In this review we will focus on the canonical Wnts, as these are most associated with HCC in general and HCV-infection-associated HCC in particular.

Except for several stem cell niches, canonical Wnt/β-catenin signaling is typically not active in tissues of adult individuals [17], despite constitutive production of Wnt ligands. This is a result of the action of a range of Wnt antagonists, such as secreted frizzled (FZD)-related proteins, dickkopf, and Wnt inhibitory factor [18]. In this nonsignaling state, cytosolic β-catenin is continuously phosphorylated at Ser33, Ser37, Thr41, and Ser45 residues located in exon 3 by a multiprotein complex consisting of adenomatous polyposis coli (APC), axin, glycogen synthase kinase 3β (GSK3β), and casein kinase 1. These phosphorylations cause β-catenin to be recognized and polyubiquitinated by β-transducin repeat containing protein, followed by β-catenin degradation in the proteasome [18, 19]. The overall effect is that minimal free cytosolic β-catenin is available for nuclear signaling, and thus Wnt-mediated gene transcription is absent under normal conditions.

On binding of Wnt ligands to a complex consisting of the FZD receptor and coreceptors, which include low-density lipoprotein receptor related protein 5/6, the scaffolding protein disheveled is recruited to the membrane, an event that in turn causes the disassembly of the multiprotein β-catenin destruction complex. This results in rescue of β-catenin from proteasomal degradation and thus the accumulation of β-catenin in the cytoplasm, eventually causing β-catenin translocation to the nucleus [20]. In the nucleus, β-catenin binds transcription factors of the T-cell factor 4 (transcription factor 7, transcription factor 7 like 1, and transcription factor 7 like 2)/lymphoid enhancer binding factor family, triggering transcription of downstream Wnt target genes, including *CYCLIND1*, *AXIN2*, *MYC*, *RNF43* (which encodes ring finger protein 43, RNF43), and *ZNRF3* (which encodes zinc/ring finger protein 3, ZNRF3) [21, 22]. RNF43 and ZNRF3 are two closely related transmembrane E3 ligases, which remove surface FZD receptors by promoting their endocytosis [23]. This E3 ligase activity is in turn negatively modulated by R-spondins and leucine-rich repeat containing G-protein-coupled receptor 4/5/6, which sequestrate RNF43 and ZNRF3 from FZD receptors by forming a tripartite complex [24]. Hence regulation of Wnt target gene transcription is complex, allowing extensive regulation but also mechanisms leading to deregulation of target gene transcription in pathophysiology.

Further complexity is added by the role of β-catenin in cell–cell adhesion, where it acts, independent of its transcriptional activity, by forming a complex with cadherins and facilitating the formation of cellular junctions between adjacent hepatocytes. The β-catenin captured in these cell-adhesion complexes represents a dynamic pool of β-catenin capable of nuclear signaling following several stimuli. One of these stimuli is β-catenin tyrosine phosphorylation by receptor tyrosine kinases activated by growth factors produced by epithelial and stromal cells. In particular, phosphorylation of the β-catenin residue Tyr654 results in its release from cadherins and an increase in T-cell-factor-mediated transcriptional activity [25–28]. Furthermore, the adherence pool of β-catenin also appears to be under indirect control of Wnt signaling itself. On activation of canonical Wnt/β-catenin signaling, the suppression of GSK3β leads to the upregulation of SNAIL [29]. As SNAIL is a repressor of the *CDH1* gene encoding E-cadherin [30, 31], this will lead to reduced E-cadherin production. Diminished E-cadherin production causes the dissociation of the complex and subsequent internalization of β-catenin and accumulation of β-catenin in the perinuclear endocytic recycling compartment, which promotes translocation to the nucleus to activate Wnt/β-catenin signaling [32, 33]. Hence pathogens can also provoke β-catenin signaling by disrupting intercellular junctions, in addition to direct effects on elements of the Wnt signaling cascade involved in regulating β-catenin-mediated transcription.

## Aberrant activation of Wnt/β-catenin signaling during HCC

Important in the context of potential modulation by HCV infection in relation to HCC is that aberrant signal transduction in general and β-catenin signaling in particular is one of the key characteristics of hepatocarcinogenesis [34]. Functional deregulation of Wnt/β-catenin signaling is reported frequently in HCC, strongly suggesting that this pathway is important in this tumor type. Various genetic and molecular alterations have been identified to be pro-oncogenic in a variety of settings, and have as a common denominator that they stabilize β-catenin, thus provoking enhanced transcriptional activity of Wnt target genes. Table 1 summarizes the relative mutation frequency of Wnt/β-catenin signaling elements in HCC patients. From HCC cohorts from different countries, the most prevalent are activating mutations in *CTNNB1* (which encodes β-catenin), followed by loss-of-function mutations in *AXIN1*, *AXIN2*, and *APC*. The relative mutation frequencies of these various Wnt/β-catenin signaling elements are different in HCC as compared with other cancers (e.g., sporadic colorectal cancer). The reason that these differences emerge may result from different causes of HCC and thus the type of mutations induced in liver genomes as compared with other sites in the body, but may also derive from the fact that in different organs, optimal cancer-driving Wnt/β-catenin signaling mutations may be substantially different, resulting in selection pressure for different types of mutations [35, 36]. As indicated in Table 1, around 22.1% of HCCs harbor specific gain-of-function mutations of *CTNNB1*. Missense, insertion, or partial deletions within *CTNNB1* exon 3 lead to the generation of a mutant β-catenin preventing the proper phosphorylation of amino acids Ser33, Ser37, Thr41, and Ser45, resulting in compromised degradation and thus stabilization of β-catenin in the cytoplasm. Less frequently, loss-of-function mutation of *AXIN1*, *AXIN2*, or *APC* is found in 3.2%, 0.4%, and 0.2% of HCCs respectively, evidently contrasting with the situation in colorectal cancer, where up to 80% of cancers display mutated APC [36, 37]. Frameshift in or deletion of these genes yields impaired ability of the destruction complex to degrade β-catenin and is thus also associated with enhanced Wnt/β-catenin signaling. Overexpression of upstream ligands or cell surface receptors and reduction of expression of extracellular inhibitors have been reported to stimulate activation of this pathway in HCC as well [38]. Thus at some stage in the progression toward full-blown HCC, acquisition of increased Wnt/β-catenin signaling provides liver cancer cells with a relative advantage over cells not having such mutations. Here we will argue that especially HCV infections create the conditions that allow precarcinogenic cancer cells to exhibit such enhanced Wnt/β-catenin signaling.

**Table 1:** Genetic mutation in components of the Wnt/β-catenin pathway in hepatocellular carcinoma

References	Patients	Mutant samples				Region
		*CTNNB1*	*AXIN1*	*AXIN2*	*APC*	
Rebouissou et al. [39]	373	146 (39%)	NA	NA	NA	France, Spain, Italy
Hirotsu et al. [40]	9	2 (22.2%)	NA	NA	NA	Japan
Schulze et al. [41]	243	95 (37.4%)	27 (11.1%)	3 (1.2%)	4 (1.6%)	France, Italy, Spain
Kan et al. [42]	88	14 (15.9%)	4 (4.5%)	2 (2.3%)	2 (2.3%)	China
Kitao et al. [43]	134	27 (20.1%)	NA	NA	NA	Japan
Ding et al. [44]	156	15 (9.6%)	NA	NA	NA	China
Tornesello et al. [45]	67	10 (14.9%)	NA	NA	NA	Southern Italy
Cleary et al. [46]	87	20 (22.9%)	NA	NA	NA	Canada, USA
Guichard et al. [47]	125	41 (32.8%)	19 (15.2%)	NA	2 (1.6%)	France
Lachenmayer et al. [48]	90	29 (32.2%)	NA	NA	NA	USA, Netherlands, Italy, Spain, Germany
Li et al. [49]	139	28 (20.1%)	NA	NA	NA	USA, Netherlands, China
Cieply et al. [50]	32	9 (28.1%)	NA	NA	NA	USA
Bengochea et al. [38]	62	16 (25.8%)	NA	NA	NA	Thailand, France
Austinat et al. [51]	40	10 (25%)	2 (5%)	NA	NA	Germany
Kim et al. [52]	36	1 (2.8%)	9 (25%)	NA	NA	Korea
Zucman-Rossi et al. [53]	45	18 (40%)	5 (11.1%)	NA	NA	France
Boyault et al. [54]	120	34 (28.3%)	13 (10.8%)	NA	NA	France
Zucman-Rossi et al. [55]	96	12 (12.5%)	NA	NA	NA	France
Park et al. [56]	81	13 (16%)	5 (6.2%)	NA	NA	Korea
Ishizaki et al. [57]	89	10 (11.2%)	13 (14.6)	9 (10.1%)	NA	Japan
Cui et al. [58]	34	15 (44.1%)	NA	NA	NA	China
Edamoto et al. [59]	100	24 (24%)	NA	NA	0	Japan, Switzerland
Taniguchi et al. [60]	73	14 (19.2%)	7 (9.6%)	2 (2.7%)	NA	UK
Wong et al. [61]	60	7 (11.7%)	NA	NA	NA	China
Mao et al. [62]	262	37 (14.1%)	NA	NA	NA	Taiwan
Cui et al. [63]	34	15 (44.1%)	NA	NA	NA	China
Laurent–Puig et al. [64]	137	26 (19%)	12 (8.8%)	NA	NA	France
Devereux et al. [65]	62	5 (8.1%)	NA	NA	NA	China
Hsu et al. [66]	434	57 (13.1%)	NA	NA	NA	Taiwan
Satoh et al. [67]	87	0 (0%)	5 (5.7%)	NA	NA	Japan
Huang et al. [68]	22	9 (41%)	NA	NA	NA	Japan, Switzerland
Legoix et al. [69]	119	21 (17.6%)	NA	NA	NA	France
Terris et al. [70]	73	14 (19.2%)	NA	NA	NA	France
Kondo et al. [71]	38	9 (24%)	NA	NA	NA	Japan
Van Nhieu et al. [72]	35	12 (34.3%)	NA	NA	NA	France
Miyoshi et al. [73]	75	14 (18.7%)	NA	NA	NA	Japan
de La Coste et al. [74]	31	8 (25.8%)	NA	NA	NA	France
Total	3788	837 (22.1%)	121 (3.2%)	16 (0.4%)	8 (0.2%)	

*NA* not analyzed

## High frequency of *CTNNB1* mutation in HCV-related HCC

**Fig. 1:**
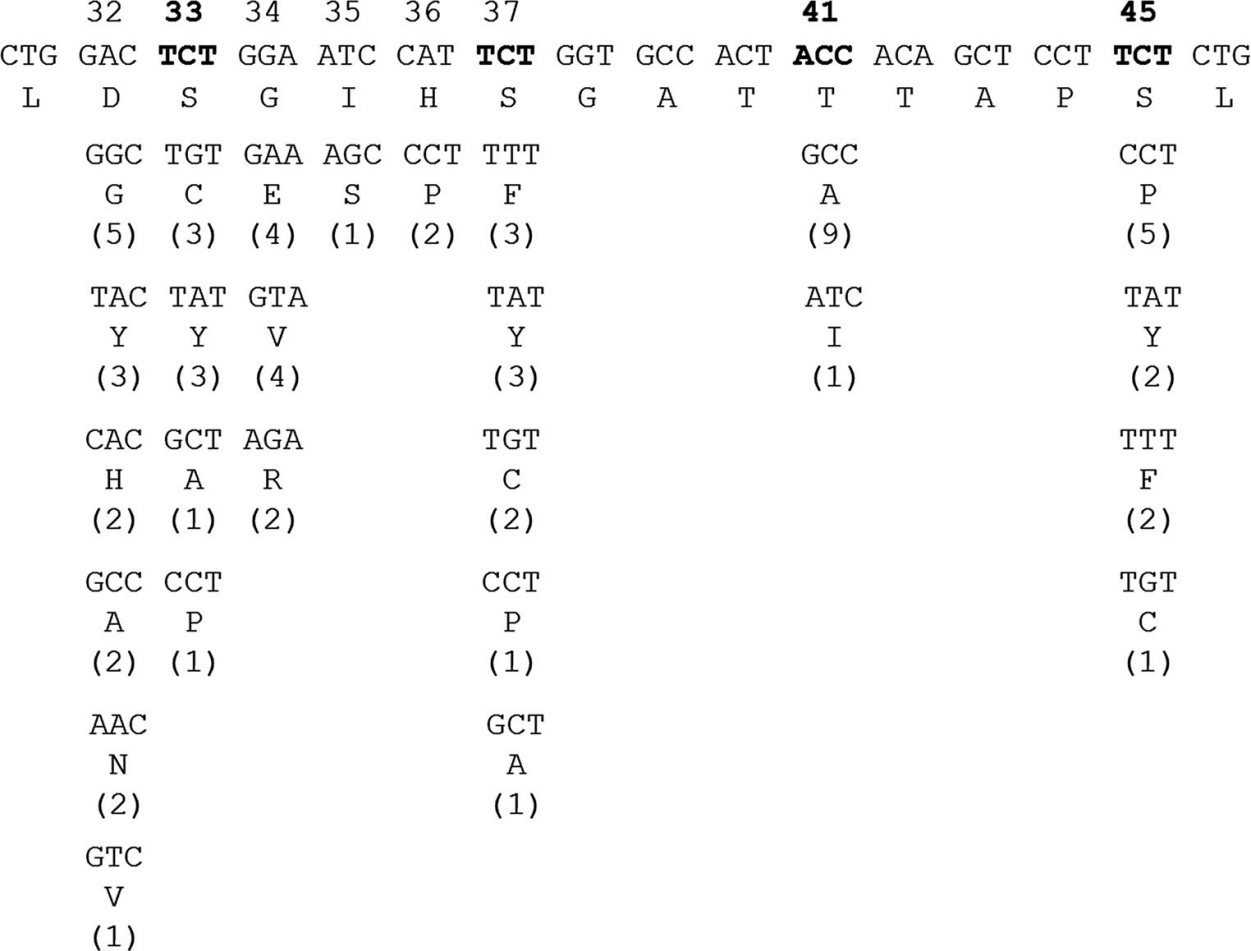
Summary of *CTNNB1* exon 3 mutations in hepatitis C virus related hepatocellular carcinoma. The locations of the *CTNNB1* mutations reported in 68 tumors from 65 hepatocellular carcinoma patients (one tumor with p.D32_G48del, not shown) are illustrated. N-terminal serine and threonine phosphorylation residues are indicated in *bold*. *Numbers in parentheses* are the absolute number of tumors tested with the given mutation

HCV infection presents a substantial clinical challenge, for which only direct antiviral medication appears to be a suitable solution [75]. If left untreated or not recognized soon enough, persistent HCV infection causes immune-mediated chronic liver damage and compensatory hepatic regeneration by inducing cell proliferation and thus creates a microenvironment permissive for the induction of genetic alterations to the hepatocyte genome [76]. Following HCV infections, genetic abnormalities accumulate relatively slowly during the sequence of chronic hepatitis and increased cirrhosis that finally progresses to HCC. Consequently, the selective growth advantage provided to hepatocytes with a malignant phenotype eventually facilitates the development of phenotypically and genetically heterogeneous HCC [77]. The relatively high frequency of mutations of *CTNNB1* (one of the principal proto-oncogenes in HCC development) in HCV-related HCC is especially striking, in the view of the relative absence of such mutations in HBV-related liver cancers but also in the view of their paucity in non-virally-associated HCC (Table 2). Around 26.7% of HCV-related HCCs harbor a *CTNNB1* mutation, which is a much higher frequency than that observed in HBV-associated HCC (11.6%) or that observed in total non-virally-associated HCC (21.2%). Furthermore, we noticed that, differently from colorectal cancers, which mainly show Thr41 and Ser45 mutations [36], HCV-related HCC shows a preference for *CTNNB1* mutations from Asp32 to Ser37 residues [45, 47, 49, 59, 68, 70, 71] (Fig. 1). Recently, a genotype–phenotype correlation was shown for *CTNNB1* mutations, suggesting that activating mutations occurring at the Asp32 to Ser37 residues lead to higher signaling levels than mutations at Thr41 and Ser45 [39]. This may partially explain the preference. It also could be attributable to the mutagenic dose required to induce HCC. Mutations at Ser45 require the selective duplication of the mutated allele as a second activating hit, whereas only one activating hit is required for mutations at Asp32 to Ser37.

**Table 2:** Comparison of *CTNNB1* mutation in subtypes of hepatocellular carcinoma

References	*CTNNB1* mutant samples			Mutation type	Amino acid	Region
	HCV	HBV	NV			
Hirotsu et al. [40]	2/5 (40%)	0/1 (0%)	0/3 (0%)	Missense	Gly34, His36	Japan
Kitao et al. [43]	12/55 (21.8%)	4/34 (11.8%)	11/44 (25%)	NA	NA	Japan
Ding et al. [44]	NA	12/110 (10.9%)	3/46 (6.5%)	Missense	Asp32, Gly34, Ser37, Thr41, Ser45	China
Tornesello et al. [45]	10/57 (17.5%)	0/10 (0%)	NA	Missense	Asp32, Ser33, Gly34 Ile35, Ser37, Ser45	Southern Italy
Kan et al. [42]	NA	12/81 (14.8%)	NA	Missense	Asp32, Ser33, Gly34 Ile35, Ser37, Thr41, Ser45	China
Guichard et al. [47]	8/24 (33.3%)	4/35 (11.4%)	30/80 (37.5%)	Missense Insertion Deletion	Asp32, Ser33, Ser37, Thr41,Thr42, Ser45	France
Li et al. [49]	14/45 (31.1%)	6/52 (11.5%)	9/44 (20.5%)	Missense Deletion	Asp32, Ser33, Gly34, His36, Ser37, Thr41, Ser45, Asn387	USA, Netherlands, China
Bengochea et al. [38]	8/20 (40%)	3/18 (16.7%)	5/24 (20.8%)	Missense Insertion	Asp32, Ser33, Ser37, Thr41 Ser45	Thailand, France
Kim et al. [52]	0/4 (0%)	0/21 (0%)	1/14 (7.1%)	Missense	Ser33	China
Park et al. [56]	0/6 (0%)	13/78 (16.7%)	NA	Missense Deletion	Asp32, Ser33, Gly34 Ile35, His36, Ser37, Thr41, Ser45	Korea
Edamoto et al. [59]	16/51 (31.4%)	5/26 (19.2%)	3/23 (13%)	Missense	Asp32, Ser33, His36, Ser37, Thr41, Ser45	Japan, Switzerland
Wong et al. [61]	0/2 (0%)	5/48 (10.4%)	2/10 (20%)	Missense Deletion	Asp32, Ser33, Gly34 Ile35, Ser37, Thr41, Ser45	China
Hsu et al. [66]	23/92 (25%)	30/323 (9.3%)	4/19 (21.1%)	Missense Deletion	Asp32, Gly34, Thr41, Ser45	Taiwan
Huang et al. [68]	9/22 (41%)	NA	NA	Missense	Asp32, Ser33, Ser37, Thr41, Ser45	Japan, Switzerland
Legoix et al. [69]	7/30 (23.3%)	5/26 (19.2%)	13/64 (20.3%)	Missense Deletion	Asp32, Ser33, Gly34, Ser37, Thr41, Ser45	France
Terris et al. [70]	2/7 (28.6)	3/14 (21.4)	9/52 (17.3)	Missense Deletion	Asp32, Ser33, Gly34, Ser37, Ser45	France
Kondo et al. [71]	7/22 (31.8%)	1/8 (12.5%)	1/9 (11.1%)	Missense Deletion	Asp32, Ser33, Gly34, Ile35, His36, Ser37, Thr41, Ser45	Japan
Total	118/442 (26.7%)	103/885 (11.6%)	91/432 (21.1%)			

*HBV* hepatitis B virus, *HCV* hepatitis C virus, *NA* not analyzed, *NV* not viral

Although *CTNNB1* mutation appears to be a late-stage event in the progression to HCC [56], the high rate of *CTNNB1* mutations observed may be directly and causally related to the HCV infectious process as in vitro studies show that both acute and chronic HCV infections provoke specifically *CTNNB1* mutations in hematological model systems and HCCs [78]. Evidently, clarification of the relationship between infection with a nonintegrating virus and subsequent *CTNNB1* mutations may prove exceedingly useful for the design of strategies aimed at preventing HCV-associated HCC.

## HCV structural proteins activate Wnt/β-catenin signaling

The HCV genome is a single-stranded positive-sense 9.6-kb RNA molecule, which includes a single open reading frame encoding a polyprotein of approximately 3000 amino acids that following translation is cleaved into ten mature proteins by both host and viral proteases. These proteins are the structural proteins (core, E1 and E2), viroporin p7, and the nonstructural proteins (NS2, NS3, NS4A, NS4B, NS5A, and NS5B). The pro-oncogenic pathogenesis of HCV appears mainly mediated by the core protein and two of the nonstructural proteins, NS3 and NS5A [79]. These pro-oncogenic effects appear to depend largely on the potential of these proteins to mediate activation of Wnt/β-catenin signaling.

### Core protein

**Fig. 2:**
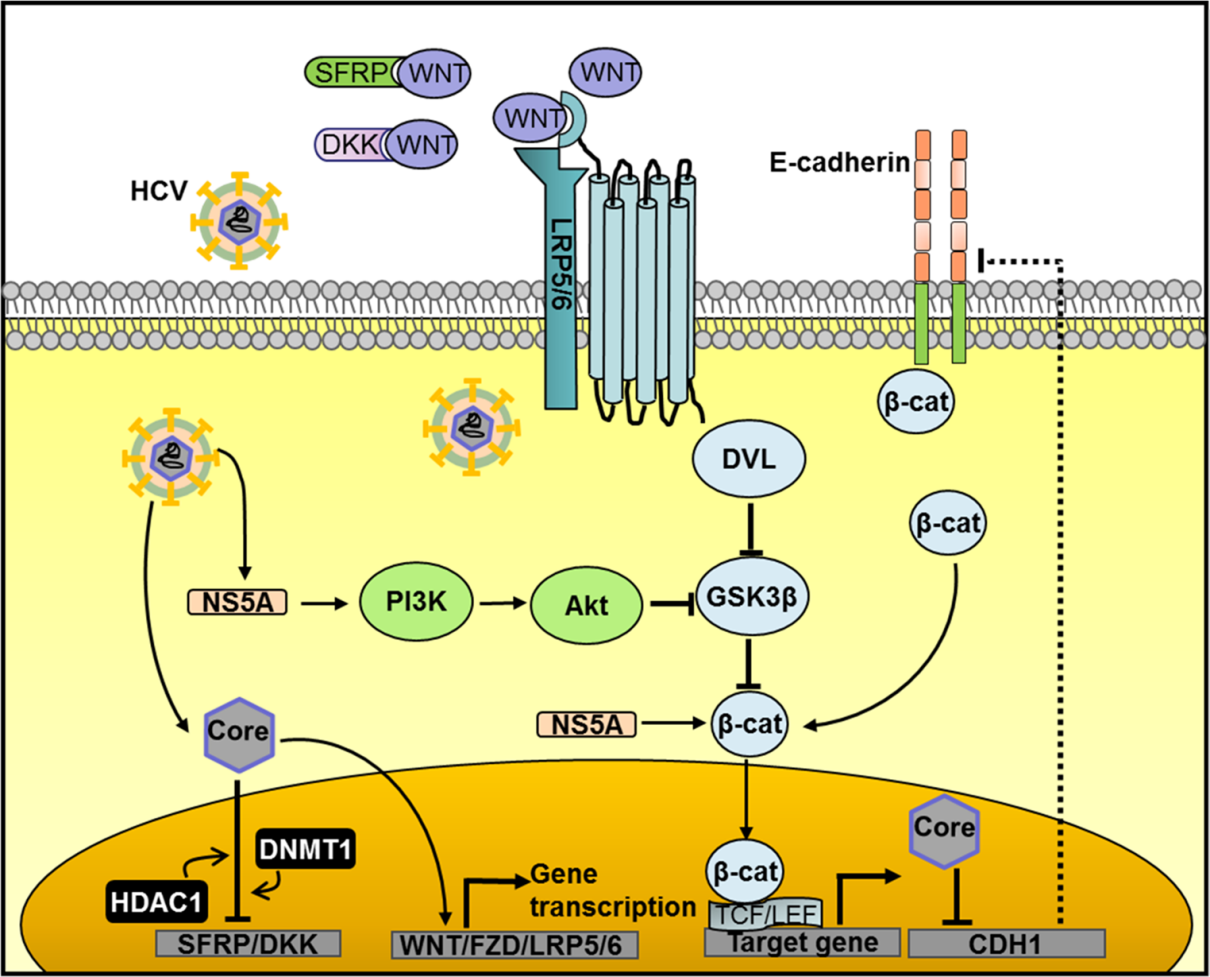
Wnt/β-catenin signaling is activated by hepatitis C virus (*HCV*) proteins. HCV core protein elevates gene expression of Wnt ligands, frizzled (*FZD*) receptor, and low-density lipoprotein receptor related protein 5/6 (*LRP5/6*) but decreases the expression of the Wnt antagonists dickkopf (*DKK*) and secreted frizzled-related protein (*SFRP*) by recruiting DNA methyltransferase 1 (*DNMT1*) and histone deacetylase 1 (*HDAC1*) to their transcription start sites. In addition, HCV core protein releases β-catenin from the β-catenin–E-cadherin complexes by suppression of the *CDH1* gene promoter (which encodes E-cadherin). NS5A protein activates phosphoinositide 3-kinase (*PI3K*)/Akt signaling, leading to the inactivation of glycogen synthase kinase 3β (*GSK3β*) and subsequent reduced breakdown of β-catenin, or directly stabilizes β-catenin. The overall effect is the cytoplasmic accumulation of β-catenin and stimulation of downstream transcription

The 21-kDa core protein is the major component of HCV. Despite lacking obvious organelle localization signals in the primary sequence, it is detected not only in the cytosol but also in the Golgi apparatus, in lipid droplets, and in the nucleus [80, 81]. Remarkably, in the latter organelle it serves as a regulator of hepatocyte transcription, facilitating Wnt/β-catenin signaling. This is brought about by upregulation of canonical Wnts, FZD receptor, and low-density lipoprotein receptor related protein 5/6 [82, 83] while concomitantly inhibiting transcription of the Wnt antagonists secreted FZD-related protein 2 and dickkopf 1 [84]. The latter effect is mediated by epigenetic silencing of the promoters involved in core-protein-mediated recruitment of DNA methyltransferase 1 and histone deacetylase 1 to the transcription start site, an effect already detected early in hepatitis infection [84, 85]. In addition, the HCV core protein mediates hypermethylation of the *CDH1* (E-cadherin) gene promoter [86]. Reduced production of E-cadherin results in diminished sequestering of β-catenin in β-catenin–E-caherin complexes and thus enhanced activation of Wnt/β-catenin signaling (Fig. 2). Hence the core protein mediates a plethora of molecular events leading to increased Wnt/β-catenin signaling and thus apparently HCV is under substantial selection pressure to provoke Wnt/β-catenin signaling. Potential sources for this selection pressure are a necessity to counteract hepatocyte apoptosis, whereas Wnt/β-catenin signaling driven expansion of the HCV-infected compartment may be involved as well.

### NS5A

The notion that HCV is under selection pressure to counteract apoptosis is further reinforced by observations that NS5A not only functions as a component of the HCV RNA replication complex [87] but also binds to the p85 regulatory subunit of phosphoinositide 3-kinase (PI3K), thus activating the downstream effector serine/threonine kinase Akt [88, 89]. Akt activation provides a powerful antiapoptotic signal and also mediates the inactivation of GSK3β, stabilization of β-catenin, and subsequent stimulation of β-catenin-dependent transcription [90]. In addition, the NS5A protein binds and stabilizes β-catenin directly [91], apparently independent of its effects on Akt and GSK3β [92] (Fig. 2). Thus the multiple stimulatory effects of NS5A on Wnt/β-catenin signaling are also testimony to the selection pressure of HCV to increase hepatocyte Wnt/β-catenin signaling.

### More Wnt/β-catenin signaling stimulating effects

The hypotheses that successful HCV infection critically depends on its potential to stimulate Wnt/β-catenin signaling is further supported by observations that, in addition to direct activation, HCV infection leads to elevation of the levels of miR-155 [93] and miR-199a-5p [94], in turn triggering Wnt/β-catenin signaling. MicroRNA miR-155 acts as an oncomiR by targeting the suppressor of the suppressor of cytokine signaling 1 gene (*SOCS1*) [95], which directly inhibits APC expression, one of the major negative regulators of Wnt/β-catenin signaling [93]. Moreover, both direct and indirect activation by HCV viral proteins may explain the notable dysregulation of Wnt/β-catenin signaling in hepatitis C and the related HCC subclass. Moreover, HCV core, NS3, and NS5A proteins may facilitate further oncogenic transformation of infected hepatocytes [79] by suppression of DNA repair mechanisms, potentially causing *CTNNB1* mutations. Support for this idea can be found in the observation that in experimental animals the hepatocarcinogenic nitrosamine diethylnitrosamine provokes cancer by inducing *CTNNB1* mutations [96, 97], and thus increased mutagenic pressure through corruption of DNA repair may be preferentially associated with this mutation. Hence the effects on the DNA repair machinery exerted by HCV core, NS3, and NS5A may link increased Wnt/β-catenin signaling mediated by direct effects of these proteins early in infection to mutation-mediated activation of Wnt/β-catenin signaling later in the progression to HCC.

## Wnt/β-catenin signaling paves the way for progression of chronic hepatitis C to HCC

### Inflammation

The HCV battles with the immune system. Thus negative modulation of inflammatory responses through enhanced Wnt/β-catenin signaling could conceivably provide further selection pressure for HCV to acquire Wnt/β-catenin signaling activating properties. However, the effect of Wnt/β-catenin signaling on hepatocyte immune responses remains controversial. On one hand, Wnt/β-catenin signaling could suppress the immune response by blunting T-cell activation [98, 99], reducing release of tumor necrosis factor [100] or stimulating the production of the chemokine-like chemotactic factor leukocyte-cell-derived chemotaxin 2 (LECT2) and invariant natural killer T cell responses, both of which relay anti-inflammatory response [101]. On the other hand, Wnt/β-catenin signaling triggers inflammatory responses by activating the proinflammatory nuclear factor κB pathway, as evident from experimentation in a hepatocyte-specific APC and LECT2 knockout (*Apc* ^−/−^ *Lect2* ^−/−^) mouse model [101]. In potential agreement, germline genetic variations in Wnt/β-catenin signaling elements were significantly associated with the risk of inflammation in HCV-infected male patients [102]. Thus the issue as to how HCV-elicited Wnt/β-catenin signaling relates to HCV-provoked inflammation warrants further experimentation.

### Fibrosis to cirrhosis and HCC development

Chronic inflammation evoked by HCV infection may culminate in liver fibrosis. Such fibrosis progresses gradually and disrupts liver physical structure and function over the course of several decades, finally resulting in fatal diseases such as cirrhosis and HCC [103]. Given HCV-stimulation of Wnt/β-catenin signaling probably evolved to support the early phases of viral infection, emerging data suggest that Wnt/β-catenin signaling activated by HCV participates in the pathogenesis of liver fibrosis as well [102, 103], mainly by enhancing hepatic stellate cell activation and survival [104]. The subsequent progression toward full-blown HCC is a complex process involving many various signaling pathways, but especially cross talk between epidermal growth factor receptor (EGFR) signaling and fibroblast growth factor (FGF) receptor signaling and aberrant activation of Wnt/β-catenin signaling appears important here.

**Fig. 3:**
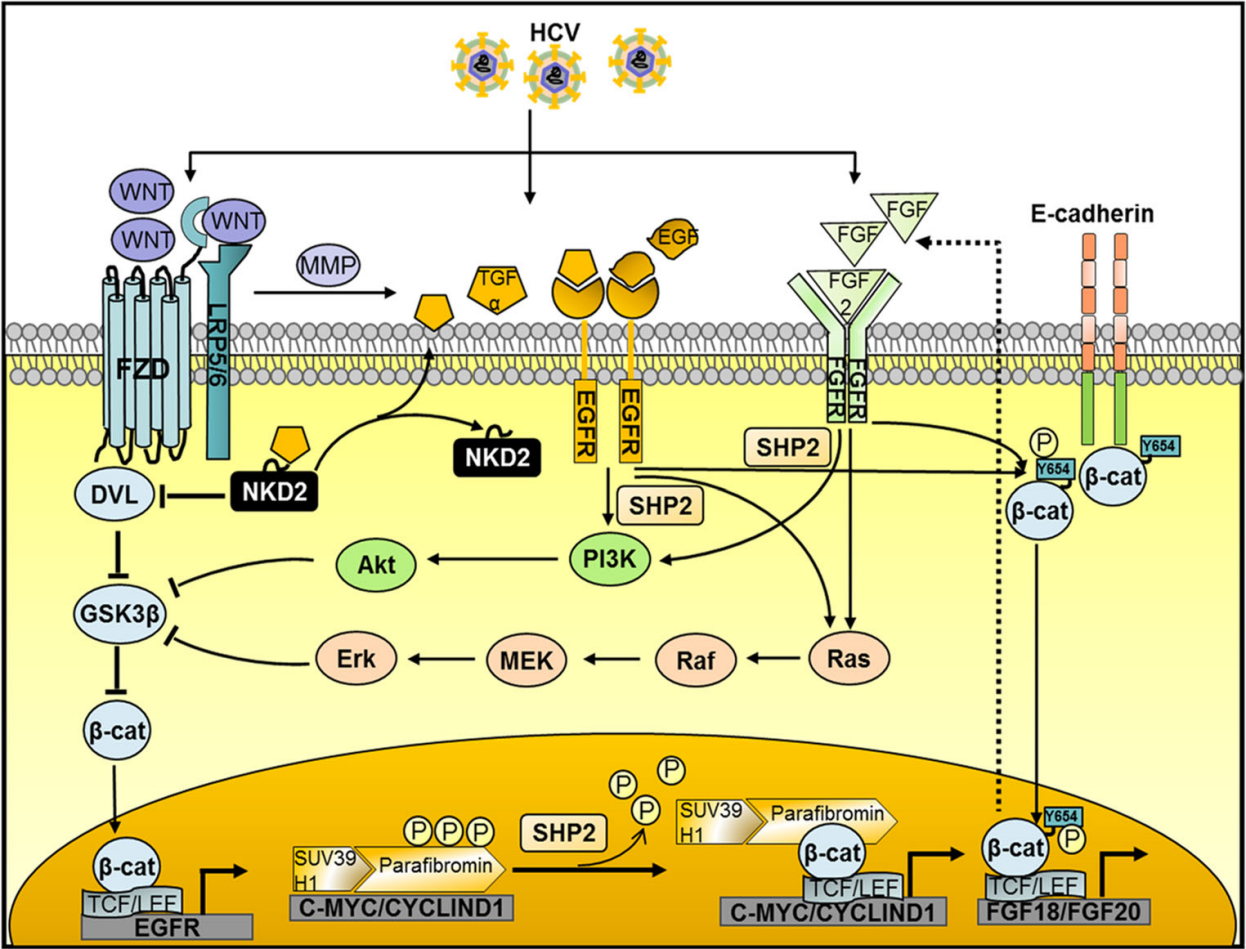
Cross talk of the Wnt/β-catenin pathway with the epidermal growth factor receptor (*EGFR*) and fibroblast growth factor (*FGF*) pathways in hepatitis C virus (*HCV*)-related hepatocellular carcinoma. HCV promotes Wnt signaling as well as the EGFR and FGF pathways. The the Wnt/β-catenin and EGFR pathways activate each other. Binding of Wnt ligands with frizzled (*FZD*) receptors transactivates EGFR signaling by matrix metalloproteinase (*MMP*)-mediated release of soluble EGFR ligands. EGFR signaling transactivates Wnt/β-catenin signaling through the phosphoinositide 3-kinase (*PI3K*)/Akt and Ras/Raf/mitogen-activated protein kinase kinase (*MEK*)/extracellular-signal-regulated kinase (*Erk*) pathways but also by releasing β-catenin from β-catenin–E-cadherin complexes as a result of Tyr654 phosphorylation. Activated β-catenin forms heterodimers with EGFR and in turn promotes the EGFR pathway. On the other hand, Wnt signaling stimulates FGF signaling by inducing FGF18 and FGF20 ligand expression. In turn, the association of FGF19 with FGFR leads to the release of β-catenin from the β-catenin–E-cadherin complexes. FGF2 signaling inhibits glycogen synthase kinase 3β (*GSK3β*) activity through the PI3K/Akt and Ras/Raf/MEK/Erk pathways. Activated Src homology region 2 domain containing phosphatase 2 (*SHP2*) in the PI3K/Akt pathway and the Ras/Raf/MEK/Erk pathway dephosphorylates parafibromin, which acquires the ability to bind β-catenin stably, overriding the repression effect on *CYCLIND1* and *MYC* expression and triggering downstream signaling

The EGFR pathway controls a variety of signals ranging from cell proliferation, cell motility, and apoptosis decrease to epithelial–mesenchymal transition, upregulation of matrix metalloproteinases (MMPs), and even stem cell maintenance [105]. EGFR is highly expressed in the adult liver [106] and plays an essential role in the G_1_–S phase transition for hepatocyte proliferation [107]. EGFR pathway dysregulation has been reported in 60–80% of HCC patients [108], and is associated with the late stages and the degree of tumor differentiation [109, 110]. EGFR favors HCV entry through co-internalization of an HCV–CD81–EGFR complex following binding of EGFR ligands to the receptor and subsequent endocytosis [111, 112]. Following clathrin-mediated endocytosis of EGFR, EGFR is routed for eventual intracellular degradation [113]. The viral NS5A protein, however, perturbs EGFR trafficking and degradation, increasing EGFR signaling and contributing to HCV-mediated HCC development [114]. Binding of Wnt1 and Wnt5a to FZD transactivates EGFR signaling by MMP-mediated release of soluble EGFR ligands, such as transforming growth factor α [115]. Activated β-catenin might form heterodimers with EGFR to enhance EGFR pathway activation [116]. Conversely, EGFR signaling contributes to Wnt/β-catenin signaling in various ways. Firstly, EGFR can directly induce tyrosine phosphorylation of β-catenin at residue Tyr654, thereby decreasing the binding with cell-adhesion complexes and releasing it for nuclear signaling [104, 117]. This phenomenon has been observed for a large number of growth factors signaling through receptor tyrosine kinases, such as hepatocyte growth factor and FGFs that are produced in excess by the cirrhotic tissue adjacent to tumor tissue [28, 118–120]. Secondly, EGFR stimulates the PI3K/Akt and Ras/Raf/mitogen-activated protein kinase kinase (MEK)/extracellular-signal-regulated kinase (ERK) cascades that both can promote β-catenin signaling through inhibition of GSK3β activity [121–125] (Fig. 3). Thus HCV-mediated activation of Wnt/β-catenin signaling may initiate a vicious interaction between EGFR and Wnt signaling, promoting potentially pro-oncogenic hepatocyte proliferation.

Similarly to the EGFR pathway, FGF-initiated signaling is a cardinal regulator of hepatocyte proliferation, differentiation, embryonic development, and organogenesis as well as hepatic tumorigenesis [126, 127]. Especially in chronic hepatitis C associated HCC, activation of FGF signaling is observed [128, 129] and increased FGF levels are associated with enhanced HCV replication and release of infectious particles [130]. Cross talk of Wnt and FGF pathways in HCV-related HCC is supported by observations that FGF signaling leads to the release of β-catenin from the β-catenin–E-cadherin complexes because of the phosphorylation of Tyr654 as described above. Furthermore, FGF2 increases expression of β-catenin messenger RNA, upregulates β-catenin nuclear translocation, and inactivates GSK3β [131], probably mediated through activation of the PI3K/Akt and Ras/Raf/MEK/ERK pathways. Conversely, Wnt/β-catenin signaling is able to activate FGF signaling by increasing *FGF18* and *FGF20* expression [132] (Fig. 3). Thus again, vicious interaction between Wnt/β-catenin signaling and FGF signaling appears to occur.

It has been reported that the Src homology region 2 domain containing phosphatase 2 (SHP-2) can be activated by HCV structural protein E2 [133]. Thus conceivably SHP-2 may be an effector of EGFR and FGF signaling in HCV-related HCC. Overexpression of SHP-2 promotes liver tumor cell growth and metastasis by coordinately activating not only the PI3K/Akt and Ras/Raf/MEK/ERK pathways [121] but also Wnt/β-catenin signaling [134]. The latter effect is due to tyrosine dephosphorylation of parafibromin (encoded by *CDC73*), acting as a tumor suppressor inhibiting *CYCLIND1* and *MYC*, together with suppressor of variegation 3-9 homolog 1. As a result, parafibromin acquires the ability to bind β-catenin stably, overriding the repression effect and inducing the expression of Wnt target genes [134] (Fig. 3). Together, these results suggest that SHP-2 is one of the critical molecules whose expression is enhanced during early HCV infection and contributes to the later progression to final HCC, which needs further investigation.

## Conclusion

As one of the important cascades involved in HCV-related HCC initiation and development, Wnt/β-catenin signaling is aberrantly activated by HCV viral core and NS5A proteins. In turn, stimulated Wnt/β-catenin signaling promotes progression of hepatitis C during inflammation and fibrosis, eventually promoting cirrhosis and HCC. This interaction is further aggravated by a vicious circle involving the EGFR and FGF pathways.

## Abbreviations

APC: Adenomatous polyposis coli
EGFR: Epidermal growth factor receptor
ERK: Extracellular-signal-regulated kinase
FGF: Fibroblast growth factor
FZD: Frizzled
GSK3β: Glycogen synthase kinase 3β
HBV: Hepatitis B virus
HCC: Hepatocellular carcinoma
HCV: Hepatitis C virus
LECT2: Leukocyte-cell-derived chemotaxin 2
MEK: Mitogen-activated protein kinase kinase
MMP: Matrix metalloproteinase
PI3K: Phosphoinositide 3-kinase
RNF43: Ring finger protein 43
SHP-2: Src homology region 2 domain containing phosphatase 2
ZNRF3: Zinc/ring finger protein 3
